# International Dental Federation

**Published:** 1902-03

**Authors:** 


### INTERNATIONAL DENTAL FEDERATION.

(Continued from page 135.)

### INTERNATIONAL COMMISSION OF EDUCATION.

*London, August 5, 1901.*

THE meeting was called to order at 5.15 P.M. by Dr. Godon, president of the International Dental Federation.

The following members answered the roll-call: Drs. Brophy, Bryan, Aguilar, Godon, Sauvez, Queudot, Viau, Roy, Harlan, Kirk, Förberg, Haderup, Zsigmondy, Frank, Baruch, Quartermann, Huet, Hesse, and Weiser.

Dr. Förberg presented, on behalf of Dr. Sandstedt, of Stockholm, excuses for the absence of the latter.

Dr. Godon said that the commission would proceed to the election of officers for the present session, and that at the last meeting the commission would decide whether the officers elected should continue in office for another year or not.

Dr. Aguilar proposed the appointing of a committee whose duty should be to nominate the officers.

Dr. Godon made a motion to the effect that the officers of the International Dental Federation compose that committee. Carried.

During the absence of Dr. Godon (who left the meeting for a short time with the officers of the International Dental Federation in order to discuss the question of nominating the officers of the International Commission of Education) Dr. Frank occupied the chair.

Dr. Brophy then opened the general discussion upon Dental Education.

The following report was then read by Dr. Zsigmondy:

REPORT ON DENTAL EDUCATION (BY DRS. ZSIGMONDY AND WEISER).

In Austria, dentistry has always been considered as a part of surgery. In spite of the defects and errors of the law, and also notwithstanding the negligence of the government in its application, this law has always required that the dentist shall have completed his medical studies before being allowed to practise dental surgery. The physicians in Austria do not want this condition of things changed. Even by making the greatest concession and fully acknowledging the modern progress, it cannot be admitted that dentistry is composed of two-thirds of manual training and one-third of surgical science. We see every day in our practice that this third is a very important factor in dentistry. We also see that it is not very difficult for a physician to become familiar with the technical two-thirds of this art, while, on the contrary, it is very difficult for the dental mechanic to acquire the said one-third of surgical science necessary for the treating of diseases consecutive to disturbances of the teeth,—that is, diseases of the gums, of the jaws, of the maxillary sinus; also to possess himself of the necessary knowledge required for the administration of general and local anæsthesia. It is absolutely indispensable that a dentist should know the course to be followed in cases of this sort, for incertitude may have very disastrous effects. During the last ten years we find more than one antagonist to this opinion, on various grounds, and in support of their arguments against the study of medicine by the dentist they claim that in order to become a good dentist manual training should be begun at the age of fourteen, or eighteen at the latest. This argument is contestable in itself, and also because the contrary is every day in evidence. It is contestable because every one who intends to follow the surgical profession must possess a certain degree of dexterity and manual training, and it is certain that this dexterity will be increased through anatomical studies, the making of microscopical preparations, the use of chemical and physical appliances, also by operations upon living and dead subjects. This refutes the fact that the number of Austrian *médecin-dentistes* capable of making their own prosthetic work is becoming more limited every day. It is also refuted by the scientific literature of ancient date, in which we find important works by Austro-Hungarian writers, just as at the present time our authors have acquired a reputation in this branch of dental literature. We see that in Austria reviews are published composed principally of papers by Austrian writers. This comparatively great development has taken place in spite of the fact that we have only three clinics or dental courses poorly endowed by the state (and this only very recently) and one supported by private funds.

By comparing this development with the erroneous position in which dentistry finds itself to-day in the countries where this part of medical science has not been or is not protected by appropriate laws and by good special schools, but on the contrary is open to any one, it can be easily seen that this result has been attained only by the fact that in Austria dentistry has always been in the hands of physicians who do not spare any time, work, or material sacrifice in order to improve their manual abilities, either by means of special studies or else by taking courses at foreign colleges.

The *médecin-dentistes* of Austria observe with satisfaction that their art has attained in their country the same degree of perfection as in the majority of the other states of Europe, and this notwithstanding the limited number of special clinics and their small endowments. By the new method of examinations of the medical school, which becomes legal in 1903, dentistry will be a compulsory study, and the government having promised the organization of new clinics and new courses in all our universities, this progress will take considerable proportions,—in fact, the development of medical science will lead necessarily towards a complete reorganization of the study of medicine. It is inevitable that the students shall take up at first and during three or four years the study of the general and theoretical branches of this science, general and pathological anatomy, physics, chemistry, physiology, general pathology, microscopy, bacteriology, internal medicine, also a second course of about two years, which would be devoted to the special branches of medicine. Nevertheless, it should not be thought that because we are optimistic we are also blind to the defects of the method of examination of our schools of medicine, defects that cannot be denied, any more than can the errors of our legislature with regard to this subject,—that is, that the degree of dentist is not absolutely connected with examinations and with sufficient proofs of special studies in this important branch of medical science. The corporations of *médecin-dentistes* of Austria-Hungary many years ago made fruitless efforts to bring about a change as to this, but the government has remained indifferent, because it does not attach enough importance to it, and also because it does not want to go into the expense of organizing new courses and new clinics; but if this effort has not yet succeeded, we hope that the official representatives newly created by the "Aerztekammer," and particularly by the "Organisation der Aerzte," will be more successful. At the present time they are working with the government to the end that doctors of general medicine who have given proofs of their aptitude by means of special studies and special examination shall alone have the right to practise dentistry. The dentists of Austria are persuaded that this is the only way of putting an end to the controversies between the *médecin-dentistes* and the technical dentist. They hope this inasmuch as in Italy and other countries it is also desired that the dentists shall be doctors of medicine, and in Germany a congress of professors of dentistry has been organized in order to ask the government that the study of this part of medical science shall be accessible only to students that have passed their baccalaureate degree. This congress made a petition to the Council concerning changes in the examinations by which the studies and examinations in dentistry shall be made to resemble the studies and examinations in medicine. The Austrian dentists are of the opinion that it is their duty to defend and improve the system in vogue in their country, which looks upon dentistry as a specialty of medicine.

*Dr. Haderup.*—We have just heard the exposition of two perfectly opposite principles. The Austrian representatives believe that dental education should have for its basis a complete medical education, while Dr. Brophy, of Chicago, on the contrary, asks that the future dentist should be first of all a practitioner and that he should receive his complete dental education in a dental school.

Before giving you my opinion on this capital question, I want to explain my position. I began the practice of dentistry twenty-four years ago; at that time I was a physician, and my opinion on dental education was similar to that of the Austrian representatives, but at the present time my opinion is different, and for thirteen years I have believed that complete dental education should be given in a special school. The experience that I have been able to gather in this direction has also confirmed my opinion that the dental students should only take up the medical studies necessary for their special calling, and that the theoretical and practical instruction should work side by side and should be given as far as possible in the dental school itself.

As far as the essential points of this problem are concerned I must say that I agree with Dr. Brophy's observations; however, he seems to me to have a tendency to consider all the branches of dental education as being of a purely practical nature, and to accord only a very small degree of importance to the theoretical subjects.

He is right if he refers to the sterile theories which are in no way connected with the teaching of the practical subjects, but my personal opinion is that the dentist cannot do without the scientific knowledge which forms the basis of practical education in all walks of life. In one word, dental education must be not only practical, but theoretico-practical.

Dr. Godon at this point resumed the chair and announced that the committee had made the following nominations: For president, Dr. Brophy; for vice-presidents, Drs. Zsigmondy, Kirk, and Paterson.

The meeting then proceeded to vote upon these names. The gentlemen whose names were proposed were elected to their respective offices.

*London, August 6, 1901.*

The meeting was called to order at 10.45 A.M. by President Brophy, the following members being present: Drs. Brophy,

Aguilar, Bryan, Hesse, Zsigmondy, Weiser, Baruch, Frank, Huet, Quartermann, Queudot, Sauvez, Godon, Viau, and Roy.

Dr. Sauvez announced that the Executive Council had appointed Drs. Bryan, Haderup, and Zsigmondy members of the Commission of Education. He then explained Dr. Godon's motive in having at first resigned his office in the International Dental Federation, which was that the position vacated might be open to the other nationalities.

Dr. Brophy then announced that the next thing on the programme was the continuation of the discussion upon dental education.

Dr. Aguilar said that he was very happy to see the organization of the International Commission of Education according to the resolution that he presented to the Congress last year. Replying to Dr. Brophy, he said that his opinions (Dr. Brophy's) could be applied to the United States and to countries where dental education is organized, but that in the countries where dental education has not as yet been organized they are not applicable, as in such a case nothing can be done without the help of the government, and hence the intervention of the university cannot be avoided. Dr. Aguilar said that the general education of the dentist should be similar to that of the physician, and hence the scientific and medical branches should be taught in the schools of medicine, which are the most competent ones for this purpose.

He asked that the commission should determine the subjects that dental education should comprise.

Dr. Hesse said that the question of how the subjects should be taught should not be taken into consideration, and that instead it should be decided what subjects should be included in the dental curriculum.

*Dr. Roy.*—This discussion is a very important one, as opportunity is given to us to hear the views of the representatives of two opposite parties,—those who claim a medical education for the dentist, and those who are of the opinion that to the dentist a special education should be given. The controversy between these two parties is not a new one, as we find it at the beginning of the organization of dental education. The first dental school, the Baltimore College of Dental Surgery, was founded only after many unsuccessful attempts to institute the teaching of dentistry in the medical schools. It was the foundation of this school that initiated the autonomy of dentistry. This difference between the two principles, the medical principle and the odontological principle, is not only a very ancient but also a very general one, and is found in all countries to a greater or less degree.

What are the reasons for the sometimes very warm controversies between people who are animated with the same ideal,—the progress of the profession? The reasons are to be found in the special features characteristic of dentistry, which is a mixed profession, and in the fundamental nature of the problem calling for decision,—viz., whether dental education should take for its basis the medical studies, or whether the dental studies should constitute an autonomous education.

Those who have medical tendencies think that dentistry is a branch of medicine to the same degree as ophthalmology or laryngology. For them, consequently, the study of medicine represents the best preparation for the practice of this specialty, the distinctly practical subjects being acquired very quickly. Following this line of thought, Dr. Arkövy told us yesterday that six months of special study is all that is required. Those of the opposite party, on the contrary, proclaim the autonomy of the profession. While acknowledging that dentistry has some points in common with medicine, such mutual borrowings as are frequent between the sciences, they claim that skill plays a very important *rôle* in the practice of dentistry; that this skill takes time and is difficult to acquire, and that it is not very much to devote to it a few years of progressive training of the eye and hand.

Hence, I think that the whole problem resides in this difference of appreciation of the time necessary to acquire manual skill. Some think that this should be of short duration; others, on the other hand, that it should be of long duration. There is, in fact, a point that should be established at the beginning of this discussion, and this is that it is not possible in four or five years to become both a good physician and a good dentist; it takes almost ten years. But such a long period of study is impossible to require either from the medical or from the dental student. The result of such a measure would mean the diminution in the number of dentists, and this I think would prove to be most detrimental in the sense that those with limited means could not have their teeth attended to, as the diminution in the number of dentists would result in the raising of fees. All classes of society should be able to profit by the progress made by dental therapeutics, and I do not agree with Dr. Guillermin, who, in the report that he read yesterday, complains of the large number of dentists and of the tendency to use charlatanic proceedings which are detrimental to the dental profesion and which are brought about by this accumulation of dentists.

The medical degree for the dentist is only an exception. Note the conditions existing in Austria, where this diploma is required; there is, in spite of all, a special group, the Zahnkünstler, which make a competition to the *médecin-dentistes,* naturally brought about by the reasons I have pointed out to you, and if the Zahnkünstler exist it is because they answer an existing need. Hence, as I think I have proved that a too long period of time cannot be demanded (four or five years seems to me to be the maximum that could be asked), it is indispensable to make an appropriate division between the theoretical and practical teaching. It can be seen by the data in my report to the Paris Congress, quoted by Dr. Arkövy and erroneously attributed to my friend, Dr. Godon, that in the majority of dental schools the number of hours devoted to theory is considerably inferior to those devoted to practical work.

Nevertheless, Dr. Arkövy and myself differ altogether with regard to the division of time. In order to oppose the propositions that I have furnished he has calculated the number of hours devoted in the European universities to medical studies. He found in the University of Budapest (and the same is the case for the other universities) that in five years the number of hours devoted to study amounted in 8632, and that of these 5002 are devoted to theory and 3630 to practice. He then says, "Hence, for many centuries the physicians have been badly educated!—for, contrary to what takes place in the dental schools, they have devoted more time to the theoretical studies." But Dr. Arkövy forgets just one thing, and this is that the object of the medical schools is to make physicians, and that medicine is a profession which requires the minimum amount of manual skill. If the medical schools, on the contrary, had only to educate surgeons, they would have to modify those plans by giving a greater amount of importance to the practical subjects. This is what is done in the case of those medical students who in the future will take up surgery exclusively. They devote more time (than do those who will take up medicine) to anatomical work and to operative medicine, and this with the object of increasing their manual skill. I do not want to prolong this discussion, but I think that the principle should be established that dentistry is undoubtedly a scientific profession and also a handicraft, and we need not feel ashamed of it, as does Dr. Arkövy. Hence, if it is a handicraft, it requires, as any other trade, a prolonged apprenticeship of the hand and eye, and in order not to prolong the period of study the greatest part of this should be devoted to the practical studies.

It is understood that theory should not be neglected before beginning and during the practical studies, but then the practical subjects should never be neglected, for without it good dental therapeutics (the final purpose of odontology) is not possible.

*Dr. Godon.*—The discussion demands that the commission should not study those points which bring about controversy, but those questions upon which it is easy to agree, and particularly the programme of dental education. For this purpose it would be advisable to take up the five points as brought forward by Dr. Sauvez in his report. These points were as follows: (1) Preliminary education. (2) Duration of the course and the order in which the different subjects shall be taken up. (3) Programme of the scientific and medical education. (4) Programme of the technical teaching. (5) Qualification and diploma.

He would propose the appointment of five members for the separate study of every one of these questions, the individual reports to be presented at the next session of the commission in 1902. He made a motion to the effect that the members be appointed at the next meeting at Cambridge. Carried.

Dr. Brophy asked how many countries are represented in the Federation, and was told that sixteen countries were represented, and that twelve had sent delegates.

Dr. Brophy expressed a desire that there might be representatives of all countries and schools. He said that he regretted the absence of English members in the commission.

Dr. Godon explained that Dr. Cunningham and Dr. Paterson are members of the commission representing England, but that the latter, on account of his position of secretary of the British Dental Association, had not been able to attend the meetings regularly, and that it was not possible to make an official invitation to the British Dental Association on account of difficulties met with in the organization of the new branches.

He then said that all the members would now agree in asking President Brophy to inform the British Dental Association at this afternoon's meeting of what has been done up to the present, to excuse the commission for not having addressed an official invitation to the British Dental Association, the commission being unable to do so on account of not being completely organized, and to invite the members of that Association to take part in our future meetings.

It was decided to hold the next meeting in Cambridge August 7, at eleven A.M.

*Cambridge, August 7, 1901.*

The meeting was called to order at Trinity College Hall, Cambridge, at eleven A.M. by President Brophy.

The following members were present: Drs. Brophy, Godon, Bryan, Pearson, Queudot, Viau, Aguilar, Kirk, Grevers, Zsigmondy, Weiser, Frank, Quartermann, Baruch, Hesse, Huet, Haderup, Paterson, Rosenthal, Sauvez, Harlan, and Roy. The following were absent: Drs. Förberg and Cunningham.

Dr. Roy, the secretary, read the report of the previous meeting, which was accepted after some remarks by Drs. Aguilar and Zsigmondy.

Dr. Sauvez announced that the committee of the International Dental Federation had appointed Dr. Pearson adjunct member for the present meeting.

Dr. Godon announced that two new propositions had been received from Dr. Sauvez and one from Dr. Roy.

Dr. Sauvez's proposition reads as follows:

The delegates of each country represented will be requested to prepare for next year's meeting a *résumé* of a few pages describing without any commentary the dental legislation and education of their respective countries.

The Commission would print these reports.

In the case of countries being represented by more than one delegate the member who should write the report would be appointed by the various delegates.

After a few remarks by Drs. Aguliar, Quartermann, Paterson, and Roy this proposition was adopted.

Dr. Roy's proposition reads as follows:

The Commission shall appoint a member to study the conditions of affiliation of all the schools to the International Dental Federation, and also the means of bringing about such affiliation, so that the Commission shall be in a position next year to adopt appropriate resolutions.

Dr. Godon said that this proposition was embodied in Dr. Spaulding's motion to the International Dental Congress, and proposed that the two propositions shall be combined in one.

Dr. Rosenthal then read Dr. Spaulding's proposition, and both propositions were adopted. Dr. Rosenthal was appointed to study and report upon these subjects.

Dr. Brophy announced that members would now be appointed to report upon the following questions proposed by Dr. Sauvez:

1. Preliminary education.
2. Time, duration, and order of studies.
3. Programme of the scientific and medical education.
4. Programme of the technical education.
5. Qualification and diploma.

Dr. Aguilar said that he was surprised to observe that his propositions had been totally ignored.

After a discussion in which Drs. Godon, Hesse, Sauvez, and Brophy took part, it was decided to take up Dr. Aguilar's proposition as the basis of the discussion.

Dr. Aguilar then read his first proposition:

That a member be appointed to report and present resolutions next year upon each of the following questions:

(1) What should be the preliminary requirements for admission into dental schools?

Dr. Godon proposed to suppress the words dental schools, which might awaken prejudices.

Dr. Rosenthal also made some remarks with regard to the changing of the wording of this proposition.

After a discussion in which Drs. Godon, Grevers, Paterson, Rosenthal, Hesse, and Aguilar took part, the proposition after some changes in its wording was adopted.

Dr. Aguilar then read his second proposition:

(2) What are the theoretical and new studies that the student should have concluded before being admitted to the practice of dentistry?

Dr. Sauvez proposed to divide the proposition into three parts:

1. Theoretical, scientific, and medical studies.
2. Theoretico-technical studies.

3. Practical studies.

Dr. Godon proposed only two divisions:

1. Scientific and medical studies.

2. Technical studies.

Drs. Weiser, Rosenthal, and Hesse asked that Dr. Sauvez's second proposition referring to the time, duration, and order of studies be the one to be adopted, as it is clearer and more precise.

Dr. Rosenthal proposed the following wording, which was endorsed by Dr. Aguilar:

(2) What shall be the composition, duration, and order of the subjects of the programme of dental studies?

The proposition as thus stated was adopted.

Dr. Aguilar read his third proposition:

(3) What part of the studies which are taught in the medical schools shall be followed by the dentist?

Dr. Kirk proposed to modify the proposition so that it might read as follows:

(3) What part of the studies such as are taught in the schools of medicine shall be followed by the dental student?

Dr. Brophy said that the term medicine is too general, and that it should be specified what part of anatomy and therapeutics should be taught to the dentist.

After observations by Dr. Grevers, Roy, Godon, Rosenthal, and Paterson, Dr. Aguilar's third proposition as modified by Dr. Kirk was then adopted.

Dr. Aguilar then read his fourth proposition:

(4) Which is the best title to be given to persons practising the therapeutics and the prosthetic treatment of the diseases of the mouth and teeth?

Dr. Hesse proposed not to consider this proposition, which seemed to him to be altogether out of place.

Dr. Roy proposed to add another proposition to those already adopted:

What shall be the composition of the boards that accord the right to practise?

This proposition was not adopted.

Dr. Rosenthal presented in the name of Dr. Bryan the following proposition:

That a commission be appointed to study the means of educating the public upon dental matters.

Dr. Godon was of the opinion that this proposition should be submitted to the Executive Council of the International Dental Federation in the same way as Dr. Cunningham's proposition was sent to that body.

Dr. Sauvez, in order to give some consideration to the proposition presented by Dr. Paterson at the beginning of the discussion, and in order to simplify the work of the Commission, made the following proposition:

Each member of the Commission of Education is requested to make a report upon the questions that have been referred to as far as it touches upon his own country.

Such report is a personal one and is not binding upon the countries represented. It should be addressed before March 1, 1902, to the secretary of the Commission who is in charge of the printing of the reports which are at that time to be addressed to each member.

This proposition was adopted.

Dr. Aguilar remarked that in view of the expenses which the work of the Commission would involve for the publication of the transactions, he would make the following proposition:

A voluntary subscription is opened between the different members of this Commission and the societies represented, so as to collect the necessary funds for the publication of the transactions of this meeting and of those that may take place later on.

Carried.

Dr. Aguilar also proposed:

That all the propositions to the Commission must be presented in writing before they can be taken up for consideration.

Carried.

Dr. Godon made a motion with regard to the extension of the powers of the present officers to the next meeting in Stockholm, which was carried.

The International Commission of Education then adjourned.—*Dental Cosmos.*

